# Lifestyle-Associated Risk Factors for Community-Acquired Methicillin-Resistant *Staphylococcus aureus* Carriage in the Netherlands: An Exploratory Hospital-Based Case-Control Study

**DOI:** 10.1371/journal.pone.0065594

**Published:** 2013-06-19

**Authors:** Miranda M. L. van Rijen, Marjolein F. Q. Kluytmans-van den Bergh, Erwin J. M. Verkade, Peter B. G. ten Ham, Beth J. Feingold, Jan A. J. W. Kluytmans, on behalf of the CAM Study Group

**Affiliations:** 1 Laboratory for Microbiology and Infection Control, Amphia Hospital, Breda, The Netherlands; 2 Amphia Academy Infectious Disease Foundation, Amphia Hospital, Breda, the Netherlands; 3 Laboratory for Medical Microbiology and Immunology, St. Elisabeth Hospital, Tilburg, The Netherlands; 4 Section Infectious Disease Control, Regional Health Authority, Leiden, The Netherlands; 5 Department of Earth and Planetary Sciences, Johns Hopkins University, Baltimore, Maryland, United States of America; 6 Department of Medical Microbiology and Infection Control, VU Medical Centre, Amsterdam, The Netherlands; Columbia University, United States of America

## Abstract

**Background:**

Community-acquired MRSA (CA-MRSA) is rapidly increasing. Currently, it is unknown which reservoirs are involved. An exploratory hospital-based case-control study was performed in sixteen Dutch hospitals to identify risk factors for CA-MRSA carriage in patients not belonging to established risk groups.

**Methods:**

Cases were in- or outpatients from sixteen Dutch hospitals, colonised or infected with MRSA without healthcare- or livestock-associated risk factors for MRSA carriage. Control subjects were patients not carrying MRSA, and hospitalised on the same ward or visited the same outpatients' clinic as the case. The presence of potential risk factors for CA-MRSA carriage was determined using a standardised questionnaire.

**Results:**

Regular consumption of poultry (OR 2⋅40; 95% CI 1⋅08–5⋅33), cattle density per municipality (OR 1⋅30; 95% CI 1⋅00–1⋅70), and sharing of scuba diving equipment (OR 2⋅93 95% CI 1⋅19–7⋅21) were found to be independently associated with CA-MRSA carriage. CA-MRSA carriage was not related to being of foreign origin.

**Conclusions:**

The observed association between the consumption of poultry and CA-MRSA carriage suggests that MRSA in the food chain may be a source for MRSA carriage in humans. Although sharing of scuba diving equipment was found to be associated with CA-MRSA carriage, the role played by skin abrasions in divers, the lack of decontamination of diving materials, or the favourable high salt content of sea water is currently unclear. The risk for MRSA MC398 carriage in areas with a high cattle density may be due to environmental contamination with MRSA MC398 or human-to-human transmission. Further studies are warranted to confirm our findings and to determine the absolute risks of MRSA acquisition associated with the factors identified.

## Introduction

Methicillin-resistant *Staphylococcus aureus* (MRSA) has emerged globally, both in healthcare- and community settings [1]. Traditionally, MRSA was considered to be a healthcare-acquired pathogen (HA-MRSA). However, approximately ten years ago, a second entity emerged, the so-called livestock-associated MRSA (LA-MRSA) [2]. Human carriage of LA-MRSA is strongly related to direct contact with pigs, veal calves, and live broilers, and the implicated strains invariably belong to multilocus sequence type clonal complex 398 [2], [3]. In addition, a recent case-control study has demonstrated that living in livestock dense areas is independently associated with an increased risk of LA-MRSA carriage [4]. Concurrent with the emergence of LA-MRSA, MRSA rates rapidly increased in individuals without known healthcare- or livestock-associated risk factors. This third entity has been referred to as community-acquired MRSA (CA-MRSA) [5], [6].

The emergence of CA-MRSA is considered to be the main reason for the recent increase in the burden of staphylococcal disease in the US [7]. Outbreaks of CA-MRSA infections have been described in more or less ‘closed’ populations, such as native Americans, men who have sex with men, jail inmates, children attending day care centres, military recruits, and contact sports participants [5]. In US hospitals, CA-MRSA has even replaced HA-MRSA as the most frequently occurring MRSA type [8]. In contrast, in the Netherlands, CA-MRSA sporadically causes infections, although the proportion of CA-MRSA in MRSA is substantial and increasing (up to 24%) [9]. At present it is unclear which reservoirs are involved in this increase.

Three European studies have found CA-MRSA infections to be associated with being of foreign origin [10]–[12]. Other risk factors, such as scuba diving, have been reported anecdotally [5], [13]. Several studies have reported the presence of MRSA in retail meat and other food products, but studies on the associated risks for the consumer have not been performed [14]–[16]. This exploratory hospital-based case-control study aims to identify risk factors for MRSA carriage in patients not belonging to established risk groups.

## Materials and Methods

### Ethics Statement

Ethical approval for the study was obtained by the medical ethics committee of the St. Elisabeth Hospital in Tilburg (NL 19489.008.07, protocol 0749, March 9^th^, 2009). All participating cases, controls and their household members completed and signed an informed consent form. For children aged below 18 years the informed consent form was completed by both parents or a legal representative.

### Study design and setting

A case-control study was conducted in patients recruited from sixteen hospitals throughout the Netherlands, i.e. three university, six teaching and seven non-teaching hospitals. Patients were recruited from July 2009 until July 2011.

### Study population

An eligible case was defined as a hospitalised patient or a patient visiting the outpatients' clinic of one of the participating hospitals, who had an MRSA positive clinical culture from any site, was aged above one year, had no previous history of MRSA colonisation or infection, were not known to have acquired their MRSA through nosocomial transmission, and had no known healthcare- or livestock-associated risk factor(s) for MRSA carriage as described in the Dutch MRSA guideline [17]. Screening for potential eligibility was performed by the infection control practitioners of the participating hospitals. All potentially eligible cases were subsequently confirmed and examined for definite eligibility by the coordinating investigator. Patients from abroad, patients living in a health care facility, and patients who had died before the MRSA positive culture result became available were classified as not eligible.

For every potentially eligible case, the last patient without a history of MRSA colonisation or infection, who was admitted to the same ward or visited the same outpatients' clinic as the case, prior to the MRSA positive culture result of the case, was selected as a potential hospital-based control patient. Potential control patients were approached and asked to participate in the study. When a control patient refused, the next to last patient was asked to participate, and so on. To confirm the MRSA negative status of control patients, nose and throat samples were taken during the home visit and cultured for the presence of MRSA. In the Netherlands, the prevalence of MRSA carriage is 0.11%, so the probability that the selected control patients were MRSA positive was negligible. The remaining criteria for eligibility were identical to those for cases. A hospital-based control group was preferred over a population-based control group. First, the potential risk factors for CA-MRSA carriage that were examined in this study might well be associated with health status and the need for hospital admission. Second, the detection of CA-MRSA in cases was unexpected per definition, i.e. CA-MRSA was detected in cultures taken for other reasons. Therefore, it was important to select control subjects from a population where the potential presence of CA-MRSA would have been detected in the same way as in cases. As cases could be either MRSA infected patients or asymptomatic MRSA carriers, control subjects were selected independently of the presence or absence of infections.

### Data collection during home visit

Cases and controls were visited at home by one of three trained interviewers. A standardised questionnaire was taken to identify risk factors for CA-MRSA carriage. Based on what was known from the available literature the following potential risk factors for CA-MRSA carriage were included: being of foreign origin, travel to a foreign country, day care centre attendance, professional contact with children, practicing sports, scuba diving, sharing diving equipment, and visiting a sauna [5], [10]–[13], [18]–[21]. Furthermore, as MRSA has been found in meat, the consumption of meat (beef, pork, poultry) and fish, and the preparation of dinner were included as potential risk factors [16], [22]. Finally, the questionnaire included questions on demographic characteristics. A separate questionnaire, including the same items, was completed for all household members who were willing to participate. A nasal swab and a throat swab were taken and cultured to confirm the MRSA status of cases and control subjects.

### Genotyping of MRSA isolates

All MRSA isolates were genotyped by multiple-locus variable number of tandem repeat analysis (MLVA) [23]. MLVA is known for its higher discriminatory power as compared to either multilocus sequence typing (MLST) or pulsed field gel electrophoresis (PFGE ) [23]. The MLVA profiles were clustered using a categorical clustering coefficient (unweighted-pair group method using arithmetic averages, UPGMA).

### Geospatial analysis

Data on municipality level variables of livestock and human population density, and residential locational were collected by geocoding cases and controls to their 6-digit postal code using geographic information system software (ArcGIS version 9·3), or when this was not sufficient, Google Earth. Municipality level data of the population, land area, numbers of chickens, pigs, and cattle were downloaded from the website of the Central Institute for Statistics (CBS) [24]. Population and livestock densities were calculated as the number of animals or persons per hectare of land in a municipality. For regression analysis, the natural logarithms of population and livestock densities were used.

### Statistical analysis

All analyses were performed using SPSS version 19.0 (SPSS Inc. Chicago, IL, USA). For continuous variables differences between groups were tested with Student's t-test or Mann-Whitney U test when applicable. For categorical variables differences between groups were tested with the Pearson Chi-square test. Univariate and multivariate odds ratios (OR) with 95% confidence intervals (CI) were estimated using logistic regression analysis. All variables that were found to be associated with CA-MRSA carriage in the univariate analysis at a p-value≤0.10 were included in the multivariate analyses. As the potential risk factors for MRSA MC398 and MRSA non-MC398 might be different, multivariate analyses were stratified based on the MLVA-complex. P-values≤0.05 were considered statistically significant. Adjusting for multiple testing was not performed as this study was designed as an exploratory study [25].

## Results

**Figure 1:**
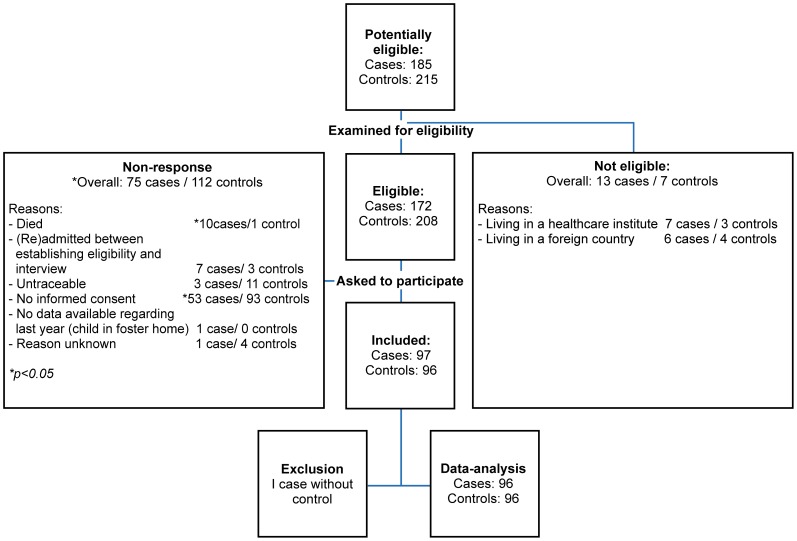
Diagram of enrolment.

185 patients were identified as potentially eligible cases based on culture results of the participating hospitals from July 2009 until June 2011. Figure 1 shows the enrolment of cases and control subjects. Ninety-seven of 172 (56%) eligible cases and 96 of 208 (46%) eligible control subjects agreed to participate and were included in the analysis. The main reasons for non-response were not providing informed consent (78%), being untraceable (7%), death (6%), and (re)admittance between establishing eligibility and the home visit (5%). Responders and non-responders were comparable with respect to age (p = 0·13), gender (p = 0·41) and social economic score (p = 0·83) (data not shown). One case was excluded from the analysis because no control subject could be found.

Baseline characteristics were comparable for cases and controls (table 1). Nose and throat samples taken during the home visit confirmed the MRSA negative status of all control subjects.

**Table 1:** Baseline characteristics.

Characteristic	Cases (n = 96)	Controls (n = 96)	p-value
*Age*			
Median – years	51.0	48.5	0.95
*Gender*			
Male – no. (%)	52 (54.2%)	52 (54.2%)	1.00
*Highest completed grade*			
No grade completed (age <11 years)	8 (8.3%)	3 (3.1%)	0.12
Elementary school	12 (12.5%)	16 (16.7%)	0.41
High school	5 (5.2%)	1 (1.0%)	0.11
Bachelor/Master degree	25 (26.1%)	23 (24.0%)	0.74
*Breadwinner*	53 (55.2%)	58 (60.4%)	0.47
*Patient type*			
Inpatient	38 (39.6%)	36 (37.5%)	0.77
Outpatient	58 (60.4%)	60 (62.5%)	0.77
*Underlying disorders*			
COPD	16 (16.7%)	16 (16.7%)	1.00
Dialysis dependent	5 (5.2%)	5 (5.2%)	1.00
Eczema	16 (16.7%)	10 (10.4%)	0.21
Insulin dependent diabetes mellitus	5 (5.2%)	2 (2.1%)	0.25
Malignancy	10 (10.4%)	7 (7.3%)	0.45

The results of univariate logistic regression analysis are shown in table 2. Consumption of poultry, at least once per week during the last year, was more common among cases than among control subjects (OR 2⋅33; 95% CI 1⋅09-5⋅00). No statistically significant differences were observed though in the consumption of pork (OR 1⋅56; 95% CI 0⋅87–2⋅82) or beef (OR 0⋅65; 95% CI 0⋅32–1⋅30). Cases had shared scuba diving equipment more often than control subjects (OR 2⋅26; 95% CI 1⋅00–5⋅14). On the other hand, cases were less likely to have practiced any sports (OR 0⋅53; 95% CI 0⋅30–0⋅94) as compared to control subjects. No statistically significant differences between cases and control subjects were found for the remaining potential risk factors, i.e. country of origin, day care centre attendance, professional contact with children, practicing contact sports, visiting a sauna, foreign travel, and livestock density. Multivariate analysis identified consumption of poultry at least once per week (OR 2⋅40, 95% CI 1⋅08–5⋅33), cattle density per municipality (OR 1⋅30, 95% CI 1⋅00–1⋅70), and sharing of scuba diving equipment (OR 2⋅93, 95% CI 1⋅19–7⋅21) as independently associated with CA-MRSA carriage (table 2).

**Table 2:** Potential risk factors for carriage of CA-MRSA.

Risk factor	Cases	Controls	Univariate		Multivariate	
	n = 96	n = 96	OR	95% CI	OR	95% CI
Born in foreign country	9	8	1.14	0.42–3.09		
Parent(s) born in foreign country	21	24	0.84	0.43–1.64		
Any foreign travel last year	50	59	0.68	0.38 1.21		
Day care centre attendance last year	3	0	∞	0.45 – ∞		
Professional contact with children last year	10	13	0.74	0.30–1.79		
Any sports last year	41	56	0.53*	0.30–0.94	0.60	0.32–1.12
Contact sports last year	5	12	0.39	0.13–1.14		
Diving last year	3	2	1.52	0.25–9.28		
Diving ever	20	14	1.54	0.73–3.27		
Sharing of diving equipment ever	20	10	2.26*	1.00–5.14	2.93**	1.19–7.21
Sauna visit last year	8	17	0.42*	0.17–1.03	0.40	0.15–1.07
Consumption of beef, ≥1× per week last year	72	79	0.65	0.32–1.30		
Consumption of pork, ≥1× per week last year	65	55	1.56	0.87–2.82		
Consumption of poultry, ≥1× per week last year	84	72	2.33*	1.09–5.00	2.40**	1.08–5.33
Consumption of fish, ≥1× per week last year	59	61	0.92	0.51–1.64		
Preparation of dinner, ≥1× per week last year	59	62	0.88	0.49–1.57		
Population density (number per hectare, natural logarithm)	1.98	2.13	0.83	0.63–1.08		
Chicken density (number per hectare, natural logarithm)	1.69	1.73	0.99	0.94–1.04		
Pig density (number per hectare, natural logarithm)	0.99	0.66	1.04	0.96–1.12		
Cattle density (number per hectare, natural logarithm)	−0.41	−0.61	1.25*	0.98–1.59	1.30**	1.00–1.70

* *p≤0·10.*

** *p≤0·05.*

MLVA typing showed that 29 (30⋅0%) of the MRSA isolates of the cases were of the MC398 type (table 3). Multivariate analyses were performed separately for cases with MRSA MC398 (n = 29) and cases with MRSA non-MC398 (n = 67) (table 4). Cattle density per municipality was found to be independently associated with MRSA MC398 carriage (OR 1·97; 95% CI 1·00–3·90), but not with MRSA non-MC398 carriage (OR 1⋅18; 95% CI 0⋅86–1⋅60). On the other hand, sharing of scuba diving equipment was independently associated with MRSA non-MC398 carriage (OR 3·90; 95% CI 1·34–11.36), but not with MRSA MC398 carriage (OR 0⋅88; 95% CI 0⋅13–6⋅22). The risk estimate of the association between the consumption of poultry and CA-MRSA carriage was comparable for MRSA MC398 carriage (OR 3·35; 95% CI 0·79–14·23) and MRSA non-MC398 carriage (OR 2·36; 95% CI 0·86–6·44), but was not statistically significant.

**Table 3:** MLVA complex and PVL data of the MRSA strains of cases.

MLVA complex	PVL positive	PVL negative	Total
	n	n	
1	0	3	3
5	5	11	16
8	5	3	8
22	1	3	4
30	9	4	13
45	1	7	8
80	3	1	4
398	0	29	29
621	1	0	1
No complex assignable	5	5	10

**Table 4:** Multivariate analysis stratified for MLVA-complex: MRSA MC398 vs. MRSA non-MC398.

Risk factor	Cases	Controls	Multivariate	
	n	n	OR	95% CI
***MRSA MC398***	***29***	***29***		
Any sports last year	8	17	0.26	0.06–1.03
Sharing of diving equipment ever	3	4	0.88	0.13–6.22
Sauna visit last year	2	7	0.51	0.07–3.94
Consumption of poultry, ≥1× per week last year	24	21	3.35	0.79–14.23
Cattle density (number per hectare, natural logarithm)	−0.09	−0.39	1.97*	1.00–3.90
***MRSA non-MC398***	***67***	***67***		
Any sports last year	33	39	0.71	0.34–1.51
Sharing of diving equipment ever	17	6	3.90*	1.34–11.36
Sauna visit last year	6	10	0.49	0.15–1.59
Consumption of poultry, ≥1× per week last year	60	51	2.36	0.86–6.44
Cattle density (number per hectare, natural logarithm)	−0.64	−0.64	1.18	0.86–1.60

* *p≤0·05.*

## Discussion

The findings of this case-control study indicate that consumption of poultry, sharing of scuba diving equipment, and cattle density per municipality are independently associated with CA-MRSA carriage. The association for sharing of scuba diving equipment was limited to MRSA non-MC398 carriage. On the other hand, cattle density per municipality was associated with MRSA MC398 carriage only. The association between poultry consumption and CA-MRSA carriage was equally strong for MRSA MC398 or MRSA non-MC398. CA-MRSA carriage was not related to being of foreign origin.

### Interpretation and implications

The finding that the regular consumption of poultry is associated with CA-MRSA carriage may be explained by the abundant presence of MRSA in meat [14], [15]. A recent study demonstrated that a substantial part of the meat pieces obtained from retail stores in the Netherlands were colonised with MRSA, including both MC398 and non-MC398 strain types. Colonisation rates varied from 10⋅6% for beef, 10⋅7% for pork, up to 16·0% for chicken and 35·3% for turkey [16]. To the best of our knowledge this study is the first to identify an association between the consumption of poultry and MRSA carriage in humans, which suggests that MRSA in the food chain may be a source for MRSA carriage in humans. The observed association might be explained by the incomplete cooking of the meat, but also by the consumption of simultaneously prepared food products, such as salads, using contaminated kitchen equipment.

In this study the sharing of scuba diving equipment was found to be independently associated with CA-MRSA carriage. This finding corresponds with previous publications of outbreaks of CA-MRSA infections in divers, and the role of sharing of scuba diving equipment as a possible mode of transmission of CA-MRSA [13], [20]. Whether the presence of skin abrasions in divers, the lack of decontamination of diving suites and other diving materials after use, or the favourable high salt content of sea water play a role in the observed association remains to be elucidated. The number of cases and control subjects that had dived the year preceding the detection of CA-MRSA is notably low, indicating that the majority of patients who had shared diving equipment acquired their CA-MRSA more than one year before the (unexpected) detection during hospital admission.

Based on the first studies that identified an association between the presence of MRSA in livestock and the occurrence of MRSA in humans, it was assumed that the risk for acquisition of MRSA MC398 was confined to persons with direct contact with livestock [2]. However, several outbreaks with MRSA MC398 in healthcare facilities have been reported since then, indicating that human-to-human transmission occurs, although it has been estimated that MRSA MC398 is 5.9 times less transmissible than MRSA non-MC398 [26]–[27]. In addition, a recently published case-control study identified pig-, cattle- and veal calf densities per municipality as independent risk factors for carriage of MRSA MC398 as compared to MRSA non-MC398 MRSA [4]. These latter finding is supported by the results of our study showing that persons without direct contact with livestock but living in areas with a high cattle density are at higher risk for MC398 MRSA carriage. Although it cannot be excluded that human-to-human transmission occurs in areas with a high MRSA MC398 pressure, environmental contamination with MRSA MC398 may play a role as well. MRSA has been shown to be present in air and soil samples collected downwind of pig and swine barns [28].

Several studies have found the presence of CA-MRSA to be associated with foreign origin [10]–[12]. The question is why being of foreign origin was not found to be associated with CA-MRSA carriage in our study. One explanation might be the selection of the study population. Where other studies have included CA-MRSA infections only, we included asymptomatic carriers as well. It might be that foreign origin is associated with the acquisition of strains that cause infection, e.g. PVL-positive strains, rather than asymptomatic carriage. In addition, previous reports may be biased by the inclusion of multiple patients from one cluster of CA-MRSA infections [10], [29]. In our study patients were derived from sixteen hospitals throughout the Netherlands. A total number of 58 different MLVA types were found, with no clustering of MLVA types, except for the livestock-associated MRSA MC398 (n = 29).

This study has several limitations. First, the study was aimed to identify risk factors for CA-MRSA, where the study population was hospital-based. This may have limited the extent of potential reservoirs to be detected as only patients were involved. Second, the participation rate was slightly higher for cases then for controls. However, as responders and non-responders were comparable with respect to age, gender, and social economic score, the potential for non-response bias is considered limited. In addition, the case definition used in this study assumes that the cases are primary cases, and does not take into account the possibility that a case might have been a secondary case, who had no exposure to the unknown source. This may have resulted in a decreased power to identify unknown risk factors. Finally, although the interviewers were intended to be blinded for the MRSA carriage status of the participants, some patients inadvertently mentioned their MRSA positivity during the interview. As a standardised questionnaire was used, the impact of this partial unblinding is expected to be limited.

The advantages of this study are the strict case definition, the acquisition of subjects from several study sites, and the performance of a home visit, which enabled thorough explanation of the questions to be completed in the questionnaire. Finally, this study was performed in a low MRSA prevalence setting (<0·1%), which allows for the identification of emerging sources [30].

### Implications for practice and further research

This case-control study identified regular consumption of poultry, living in an area with high cattle density and sharing of scuba diving materials to be independently associated with CA-MRSA carriage. However, due to the case-control study design no indication can be provided on the absolute risk of CA-MRSA carriage when either one of these exposures is present. Further studies are warranted to confirm our findings and determine the absolute risks of MRSA acquisition.
